# The intraoral device of overlaid disk-holding splints as a new in situ oral biofilm model

**DOI:** 10.4317/jced.52093

**Published:** 2015-02-01

**Authors:** Isabel Prada-López, Víctor Quintas, Inmaculada Tomás

**Affiliations:** 1 Oral Sciences Research Group. School of Medicine and Dentistry. University of Santiago de Compostela. Santiago de Compostela, Spain

## Abstract

Objectives: To design a device that allows the formation of in situ oral biofilm with similar characteristics to those from the dental plaque, overcoming the limitations of previous devices. Study Design: The Intraoral Device of Overlaid Disk-holding Splints (IDODS) was designed and manufactured. To test its validity, five healthy adult volunteers wore them for two and four days allowing the biofilm to grow without any type of distortion. After each period, the thickness, vitality and structure of the formed biofilm were measured with a Confocal Laser Scanning Microscope (CLSM) in combination with a dual fluorescence solution. All volunteers filled out a Likert-type questionnaire to evaluate the device. Results: Mean bacterial vitality in the 2- and 4-day biofilms was 71% and 63%, respectively. Mean thicknesses were 21 µm and 28 µm, respectively. There was predominance in the open and heterogeneous structure whose complexity was ascending as the biofilm matured. The results obtained from the questionnaire were 2/5 in the influence in aesthetics, 3.4/5 in comfort, and 5/5 in ease of maintaining oral hygiene and withdrawal from the oral cavity. Conclusions: A biofilm with optimum characteristics was obtained by IDODS. Its use is associated with good aesthetic and comfort results and is absent of functional limitations, allowing optimal oral hygiene without altering the structure of the in situ oral biofilm.

** Key words:**Confocal Laser Scanning Microscope, fluorochromes, in situ, intraoral device, oral biofilm.

## Introduction

The development of *in vitro* biofilm models brought significant advances in the study of oral diseases. However, due to their known limitations (1-3), the scientific community recognizes that they cannot generate a biofilm comparable to those formed *in situ*, and therefore, the results obtained in these type of studies should be interpreted with caution (2,3). From this statement, comes the need to develop models of *in situ* biofilm that can be analysed intact *ex vivo* (2).

In many studies of biofilm formed *in situ*, the sample was previously removed from the tooth surface with paper points, cotton roles or scalers for posterior analysis (4). This probably would disrupt the delicate three-dimensional relationship existing between the cells, the extracellular matrix and the substrate (5). This relationship directly influences biofilm behaviour (5), which means that for example, when an antimicrobial agent is to be studied, it is necessary to apply a method in which the three-dimensional structure of the biofilm is not distorted during the formation, the collection or analysis itself (5). Given this need, the concept of non-disturbed plaque appeared. In this type of studies, the biofilm is formed on disks of different materials that are introduced inside the oral cavity supported by specially designed devices. In the literature, several models which enable the development of non-disturbed oral biofilm are found. The first report on this issue was done by Ahrens *et al.* (6), as early as 1976. They designed a model based on acrylic splints on which enamel slides (6-8) were positioned. Since then, this system has been widely used with a variety of substrates ranging from glass (9), bovine enamel (10) and bovine dentin (11) to hydroxyapatite (12).

Another popular model has been the Leeds *in situ* device, which is a ring that includes a substrate of human enamel (5,13) or stainless steel (14) and which adheres to buccal of first and second molars using a composite resin.

Several years ago, Auschil *et al.* (2) described a new device consisting of a metal and acrylic apparatus similar to a retainer, surrounding the outline of the teeth, linking the vestibular and palatal parts, and fixing the disks with wax. The material of the disks positioned on this new device ranged from glass disks (1,2,9,15) to bovine enamel (3,16), as well as human enamel (17) and the combination of bovine enamel and titanium substrates (18).

More recently, Burgers *et al.* (19) used an apparatus consisting of individualized thermoplastic splints, on which titanium disks were fixed. This same model, with minor variations, was used by Hannig *et al.* (20) with bovine enamel and dentine fixed by silicone. Subsequently, Gu *et al.* (21) used a metal to reinforce thermoplastic soft splint where they positioned glass disks.

Although they all seem valid to achieve the success of reproducing a good quality *in situ* oral biofilm, after their careful study, all of them show limitations at some point, such as at the aesthetic and hygienic levels or the biofilm growth environment. Some of them need specific teeth for retention or a specific pre-treatment of the tooth surface (etching and bonding) with the associated problem of its potential accidental unsticking.

The aim of this study was to design an apparatus that allowed the formation of non-disturbed oral biofilm *in situ* with dental plaque-like vitality, thickness and structure, both at 2-day periods (for antimicrobial activity studies and substantivity) and four days (for studies of antiplaque activity). The design will take into account the limitations of the devices presented above.

## Material and Methods

The fabricated device was called the Intraoral Device of Overlaid Disk-holding Splints (IDODS, registered patent number: ES 2380252 B2).

To test its applicability for *in situ* analysis of the oral biofilm, a study was performed in five healthy adult volunteers (20 to 45 years) who had good oral health (with no evidence of gingivitis or periodontitis -Community Periodontal Index score = 0- or active caries). The exclusion criteria were smoking, user of removable prosthesis or orthodontic appliances, having received antibiotics routinely or used oral antiseptics in the past three months, and to have any systemic disease, metabolic state, or drug consumption that may provoke alterations in the production and/or composition of saliva. Prior to the starting of the study, all volunteers underwent a professional tooth cleaning by the same calibrated clinician. In all cases written, informed consent was obtained from participants. This study was integrated into the research project approved by the Ethics Committee for Clinical Research of Galicia, reference number 394/2012.

**Figure 1:**
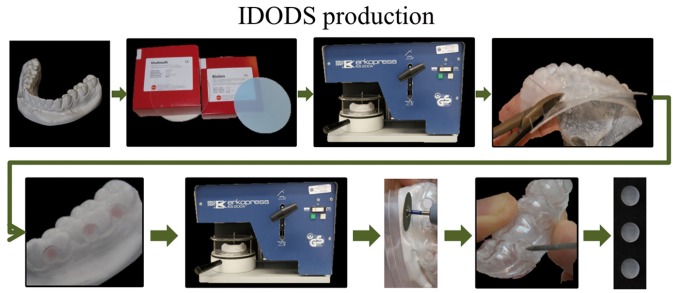
Sequence of fabrication of the Intraoral Device of Overlaid Disk-holding Splints (IDODS).

In figure 1 the IDODS manufacturing sequence is represented. Initially, a plaster model of the lower dental arch of each of the volunteers was needed. A first splint (inner sleeve) of ethylene-vinyl acetate copolymers (type Drufosoft, Dreve-Dentamid GmbH, Germany) that was soft, flexible and 1 mm thick was made on each of these models. On this splint six circular cavities of 2 mm in diameter were made; they were located in the vestibular area, between the canine-first premolar, second premolar-first molar and first molar-second molar in both hemiarches.

On the inner splint, after trimming and perforations, six manufacturing or guide disks, 6 mm in diameter and 1 mm thick, each placed over a perforation. After that, on the plaster model with the splint and the placed disks, a second splint (outer sleeve) of polyethylene terephthalate (type Biolon, CAS RN: 25038-59-9, from Dreve-Dentamid GmbH, Germany) that was rigid 1 mm thick was prepared. Thus, the outer covered the inner splint and the guide disks were housed in between them.

Subsequently, both splints were withdrawn from the plaster model to allow the removal of the guide disks and to clean up the excess of material fixing them. On the external splint, six circular cavities of 5 mm in diameter each were prepared. They were located in the same position as the perforations of the soft splint. Finally, the glass disks (on which the biofilm develops) were placed in their respective cavities, and both splints were joined with the application of heat in order to prevent any undesirable mobility during biofilm formation time.

The glass disks were lodged between the two splints, but with a surface of 5 mm exposed to buccal. This surface was protected from the action of the cheeks by an external splint frame surrounding the disk.

**Figure 2:**
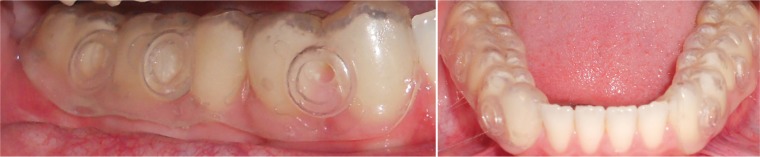
Clinical images of the “split-mouth” design of the IDODS. Note that the lower incisors are kept uncovered to improve aesthetics and comfort.

The IDODS design was modified during its use in this research. The most significant change was the ‘split-up design’ (Fig. 2). With this enhancement, the splints were more comfortable for volunteers in their normal life because their lower incisors were not covered. At the same time, this design facilitated the removal of the disks on the day of the sample analysis, preventing the unnecessary removal of the homologous splint.

Volunteers wore IDODS for two days (first test) and four days (second test), withdrawing them from the oral cavity only during meals—it was stored in an opaque container in humid conditions—and to perform oral hygiene procedures, using only mechanical removal of bacterial plaque with water without the use of any toothpaste or mouthwash.

For the analysis of the samples, a Leica TCS SPII Confocal Laser Scanning Microscope (CLSM) (Leica Microsystems Heidelber GmbH, Mannheim, Germany), was used after staining for 15 minutes in a dark chamber with the fluorescence solution LIVE/DEAD® BacLight™ (Syto 9/propidium iodide) (Molecular Probes, Leiden, The Netherlands). In the samples, parameters such as thickness, bacterial biofilm vitality and structure were analysed. For this last parameter, the maximum thickness of the biofilm (the existing perpendicular distance between the substrate and the peaks from the highest bacterial groups (16,22,23)) was divided into three layers of the same size (1 = outer layer, 2 = middle layer and 3 = inner layer). At the midpoint of each layer, the existence of the following structures was quantified in percentages: open and heterogeneous architecture, fluid-filled channels, and/or bubble-shaped structures (2,5,8,9,11). Quantification of bacterial vitality in the X-Y image series was determined by analysing the cytofluorogram (Leica Confocal Software).

At the end of both experiments, all volunteers filled up a Likert-type questionnaire in which the influence of IDODS was estimated for aesthetics, the overall feeling of comfort, how easy it was to maintain an optimal oral hygiene and the withdrawal of the device.

A descriptive analysis of the thickness, bacterial vitality percentage, and structure of the biofilm was performed. The results of the survey were presented as an average of the scores obtained by sections.

## Results

The mean value of the thickness of the 2-day samples ( Table 1) was 21.20 ± 4.09 µm. In 4-day samples, the mean thickness was 27.61 ± 2.28 µm. Regarding the bacterial vitality, the mean value for the 2-day biofilm samples was 70.62 ± 19.66%. After the evaluation of the bacterial vitality by layers, these mean values were obtained: 84.79% in layer 1; 74.27% in layer 2; and 52.80% in layer 3. In the 4-day samples, the bacterial vitality reached a mean value of 62.53 ± 15.11%. Its bacterial vitality by layers was 85.88%, 67.37%, and 34.33%, in layers 1, 2 and 3, respectively ( Table 2).

**Table 1:**
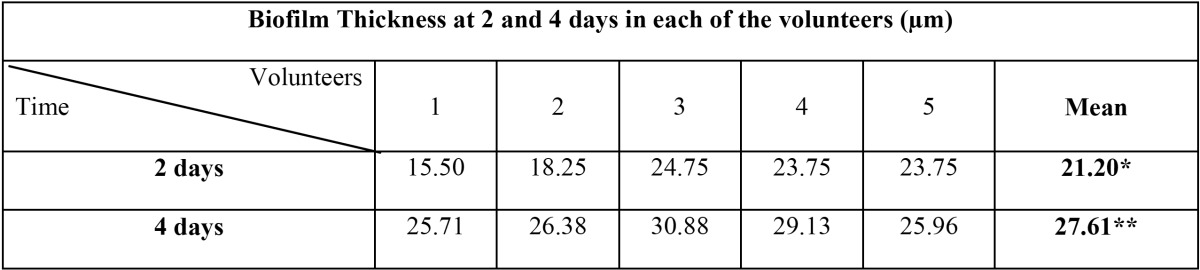
Mean thickness of the oral biofilm for each volunteer after 2 and 4 days. *SD = 4.09. **SD = 2.28.

**Table 2:**
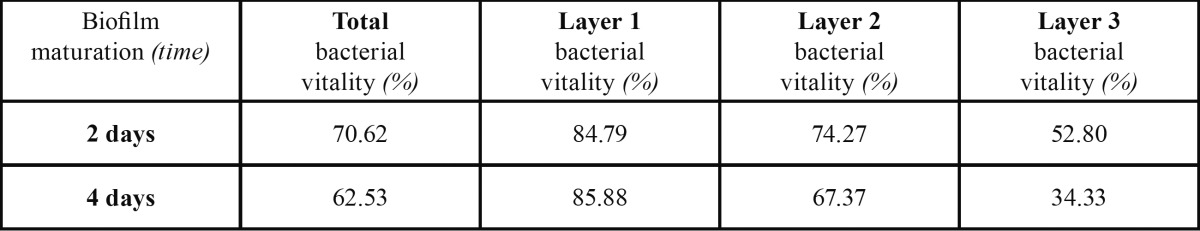
Mean bacterial vitality present in Layer 1, 2 and 3 as well as totally after 2 and 4 days of biofilm maturation.

**Figure 3:**
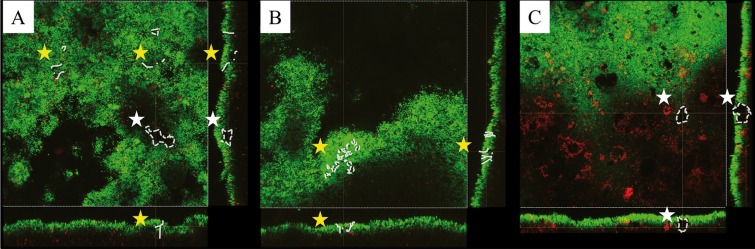
Visualization of channels (yellow stars) and voids (white stars) in different single cross sectional planes from X, Y and Z axis. Images obtained by the CLSM representing different types of biofilm structures: A) The presence of both channels and bubbles shows a heterogeneous architecture model; B) Fluid filled channels; C) Bubble-like structures.

With regard to the structure of the biofilm after two days, the model which most often appeared was the one with open and heterogeneous architecture (Fig. 3A) with a clear predominance in the outer and inner layers. In a smaller number of samples, fluid-filled channels (Fig. 3B) were found, with a clear predominance in the intermediate layer. The bubble type (Fig. 3C) structures were described in one-third of the samples, with a greater presence in the middle layer of the biofilm. The structure of the biofilm formed after four days presented an open structure in all samples, with a complex and heterogeneous architecture.

With regard to the questionnaire, the influence of IDODS in aesthetics was low (2 out of 5). With regard to the overall feeling of comfort, the score was average-high (3.4 out of 5). IDODS scored top marks by all volunteers (5 out of 5) when the ease to maintain oral hygiene and withdrawal from the oral cavity were evaluated.

## Discussion

Regarding the type of removable appliance used to collect the supragingival plaque, different designs have been proposed ranging from resin splints (6-12), the Leeds *in situ* device (5,13,14), retainers adapted to hold disks or the acrylic splints with metal frame (1-3,9,15-18), to the thermoplastic individualized splints (19,20), reinforced with metal in some cases (21). These devices allow the obtaining of a non-disturbed *in situ* oral biofilm.

The IDODS overcomes many of the limitations of previous devices. This design prevents the accidental detachment of the disks during the time they remain in the mouth and protects the disk from rubbing against the cheek or tongue, facilitating their removal by the patient without altering biofilm formation, allowing in turn, optimal oral hygiene measures. In addition, the holes in the internal splint allow a continuous flow of saliva, providing a better environment for the development of the biofilm. Another important advantage is that pre-treatment of teeth with acid and/or adhesive is not necessary in order to place the disks.

In addition to all of the advantages already named, the IDODS is built on the plaster model of the subject and allows individual adaptations or modifications not being constrained by the absence of a tooth or its anatomy. Additionally, the ability to remove the disk for its analysis without damaging the biofilm and the fact that the device permits cold disinfection allow for the apparatus to be reused in multiple experiments for the same subject. Furthermore, its simple and inexpensive fabrication allows its manufacture by the investigator, achieving good value for money.

In most studies of biofilms *in situ*, the time during which the removable device is maintained in the oral cavity primarily ranges from 24 to 48 hours (1,2,7,9,11,22,23), but depending on the biofilm to be analysed, there is a very wide timing ranging, from two hours (20) up to seven days (3,13). In the present study, IDODS was tested within two to four days in order to cover the necessary time, previously standardized, for analysis of the antibacterial immediate efficacy (as well as substantivity) and short-term anti-plaque effect.

In the present series, CLSM combined with the fluorescence solution LIVE/DEAD® BacLight™ was used. This dual solution is considered to be an appropriate technique to study the biofilm vitality *in situ* (24). Furthermore, several authors have analysed oral biofilms with the help of the CLSM, incorporating a fluorescent solution, both to analyse its structure (5,7), as well as the spatial distribution of vital and non-vital bacteria (1,8,11,16,22,23). For these reasons, the scientific community recognizes that CLSM, along with fluorescence staining solutions like LIVE/DEAD® BacLight™, is a particularly useful technique for the analysis of biofilms (24,25).

• Thickness of the oral biofilm after two and four days

In 1998, Netuschil *et al.* (7) found that the thickness of the biofilm depended on each subject and the age of the plaque, subsequent studies confirmed this large inter-individual variability (1,2,9,15). This statement has been also corroborated in this study, in which the biofilm thickness after two days ranged between 15.50 and 25.75 µm. However, Zaura-Arite *et al.* (11) found no differences between individuals hyper- and hypo-forming dental plaque, although they used a slotted model substrate to support the growth of the biofilm, which could explain the absence of differences.

In the previous literature, regarding the average values, the thickness of the 2-day biofilm was between 14 and 37 µm (3,7,11,15,21-23), which is consistent with the average value of 21.20 µm obtained in this study. However, some authors obtained thicknesses ranging between 16 and 150 µm (1,2,9). These differences may be due to the method used to quantify biofilm thickness, as some authors considered the number of layers analysed with CLSM as the maximum thickness of the biofilm. The thickness measured by this technique can be conditioned by the inclination of the substrate itself or by the position thereof in the slide, which can produce a diagonal measurement of the biofilm, producing an overestimation of the thickness. Consistent with previous authors (16,22,23), in this series the software Leica Confocal SPII was used to get the distance in microns from the substrate to the highest point of the biofilm perpendicularly, which gave a more accurate measure of the biological reality.

Although to the best of authors’ knowledge, there is no study in the literature that analyses the thickness of the biofilm formed at four days, similar studies do exist at three days that obtained values ranging between 6 and 45 µm (3,7,10). Other studies discovered that after 5 days the biofilm thickness varied between 15 and 31 µm (3,8,16). These results agree with those obtained with this device, which were 27.61 µm.

Regarding the variation in thickness over time, Al-Ahmad *et al.* (3) observed that the thickness of the biofilm increased as it ma-tured, from 14.9 µm on the first day to 49.3 µm at seven days. With IDODS, the thickness increased from 21.20 µm at two days to 27.61 µm at four days. However, it has been shown that this increase in thickness is not progressive over time (3,4).

• Bacterial vitality of the oral biofilm after two and four days

In previous *in situ* studies, the mean values of biofilm bacterial vitality after two and three days ranged between 60 and 77% (1,9,11,21-23). These results agree with those obtained in the present study (70.62%).

Some studies on 5-day biofilms set a mean bacterial vitality between 57 and 63% (16), similar to those results obtained with the IDODS for four days (62.53%). Although some authors have suggested that bacterial vitality increases with the biofilm maturation period and, consequently, its thickness (11), these studies are based on analyses that examined the biofilm from 6 to 48 hours. For this reason, they should not be considered predictive of the behaviour of the bacterial vitality of the biofilm beyond those two days. However, when it comes to more mature biofilms, they acquire greater density (5), resulting in bacteria located in the centre of large bacterial aggregates in the deeper layers where they have less oxygen and fewer substrates available, which would increase the proportion of non-vital/vital bacteria (7,8).

Some authors argue that it is difficult to establish a general pattern of distribution of bacterial vitality in the oral biofilm *in situ*, due to the fact that substantial inter-individual differences have been described (11).

Nevertheless, Arweiler *et al.* (9) suggested that the ecological environment is relatively constant and described different patterns of microbial identity. In this series, despite the great variability observed in the distribution of the bacterial vitality, a characteristic pattern was identified, with a low percentage of vitality in the layers closer to the substrate, increasing towards the higher layers. This corresponds to one of the patterns described by Arweiler *et al.* (9), in which living bacteria are superposed on the dead bacteria from the basal layer, forming a new layer of living biofilm.

Auschill *et al.* (8) found that the distribution of bacterial vitality in 5-day biofilms had a profile in which the proportion of live bacteria was lower in the layer that was nearest the substrate and increased along the Z-axis towards the central area, decreasing again towards the outer layer. Conversely, Arweiler *et al.* (16) obtained from the study of a 5-day biofilm, a pattern with lower bacterial vitality values in the deeper strata (bacterial vitality at layer 3 = 45.97%) and higher ones in the surface layers (bacterial vitality at layer 1 = 67.02%), similar to those obtained by IDODS in the present series of 4-day biofilm, confirming that bacteria close to the substrate are often metabolically inactive (5,7), as discussed above.

• Structure of the oral biofilm after two and four days

Contrary to the idea —based on *in situ* studies using electron microscopy— that biofilms have a compact architecture, analysis by CLSM confirmed a model of open and heterogeneous architecture with a complex system of channels and cavities (2,5,8,9,11). The first descriptions of the architecture of the biofilm *in situ* were those from Wood *et al.* (5), which confirmed the structural heterogeneity of a four-day biofilm in terms of distribution of cells, matrix and voids. They found, surprisingly, that all the samples had pores and fluid-filled channels. Subsequently, Dige *et al.* (15) demonstrated that these channels were surrounded by cells and extracellular matrix and colonised by a small number of bacteria. Auschill *et al.* (8) also described some non-stained cavities with bubble-morphology, presumably filled with biological substances such as exopolysaccharides and glycoproteins. In the present study, in the 2- and 4-day biofilm, there was a predominance of open and heterogeneous structure, especially in the inner and outer layers of the samples. This structure was completed with structures like bubble- and fluid-filled channels in the intermediate layer, which is consistent with the complex system of channels and cavities described by previous studies.

Coinciding with the descriptions provided by Zaura-Arite *et al.* (11) and Dige *et al.* (15) in the present series, the complexity of the biofilm increased with maturation time and thickness when the results of two and four days were compared.

• Evaluation of the IDODS made by the volunteers 

After reviewing the literature, no studies have been found in which the sensations of the volunteers at wearing the device (on which biofilm grows) were evaluated. In only two studies (2,9), the authors made sure that the volunteers did not have any problems at maintaining optimal oral hygiene when wearing acrylic splints with metal. However, it seems important to test the feel of the volunteer with the device on, in order to adapt the protocols and enhance intraoral devices.

Regarding the assessment of comfort by the volunteer, IDODS was comfortable both while in mouth and during removal. As subjects in studies which used acrylic splints with metal reported (2,16), in the present series, all volunteers were able to maintain optimal oral hygiene during experiments. However, in the case of the Leeds *in situ* device (13), due to the impossibility of with-drawal from inside the oral cavity by the volunteer, the oral hygiene level maintained during the study was negatively affected in the vicinity of the device, so as to keep a non-disturbed biofilm.

Future research on the validation of IDODS should be directed towards identifying the bacterial composition of the biofilm that grows on it, confirming the results with those obtained in samples of disturbed dental plaque. Technical improvements in IDODS should be directed towards the creation of a device that can be kept in the mouth during meals and is able to analyse the influence of diet on the characteristics of the biofilm.

Conclusions

IDODS allows the development of an *in situ* biofilm at two and four days with optimum thickness, bacterial vitality and structure characteristics, which confirms the appropriateness of its use for studies of antimicrobial activity and antiplaque efficacy in the short term.

The application of IDODS is associated with good aesthetic and comfort results and is absent of functional limitations, allowing optimal oral hygiene without altering the structure of the *in situ* oral biofilm.
